# Supplementary material for: Structural Insights on Hyp-Gly-Containing Peptides as Antiplatelet Compounds through Topomer CoMFA and CoMSIA Analysis
Source: Foods. 2023 Feb 10;12(4):777. doi: 10.3390/foods12040777 (PMC9956102; doi:10.3390/foods12040777)
Supplement: Supplementary file 1 [file foods-12-00777-s001.zip › foods-2154557-supplementary.pdf]

Table S1. The Data of Topomer CoMFA analysis. [a] The value indicates the level of contribution of the R group to the activity of the entire molecule. [b] Fr shows the fragmentation method, T-template, A-automatic fragmenting, M-manual fragmenting. [c] Actv: the observed activity (pIC_{50} value). [d] Topomer CoMFA: the predicted activity (pIC_{50} value).

Sequence	Name	R1[a]	R2[a]	R3[a]	Fr[b]	Topomer CoMFA[c]	Actv[d]
OGQ	MOLECULE	0.45	0.00	0.19	T1	0.12	0.29
OGP	MOLECULE1	0.45	0.00	-0.08	A1	0.30	0.01
GOOGPQ	MOLECULE10	0.45	-0.27	-0.25	T2	-0.32	-0.42
PGE	MOLECULE11	0.05	0.00	0.19	M	0.03	-0.11
GOPGPM	MOLECULE12	0.05	-0.27	-0.22	M	-0.73	-0.78
OG	MOLECULE13	0.45	0.00	-0.03	M	-0.15	0.06
OGE	MOLECULE14	0.45	0.00	0.19	A1	0.21	0.28
PGEOG	MOLECULE15	0.45	0.1	-0.03	T3	0.07	0.17
VGPOGPA	MOLECULE16	0.45	-0.28	-0.17	A2	-0.39	-0.35
OGOMG	MOLECULE17	0.45	0.00	-0.16	A1	-0.17	-0.07
PGPK	MOLECULE18	0.05	0.00	-0.24	M	-0.45	-0.54
PGKP	MOLECULE19	0.05	0.00	0.01	M	-0.41	-0.29
OGD	MOLECULE2	0.45	0.00	0.20	A1	0.36	0.30
PGPQ	MOLECULE20	0.05	0.00	-0.25	M	-0.55	-0.54
PGQP	MOLECULE21	0.05	0.00	0.04	M	-0.38	-0.26
PGHH	MOLECULE22	0.05	0.00	0.07	M	-0.14	-0.23
OGSA	MOLECULE23	0.45	0.00	0.05	A1	0.20	0.15
EOG	MOLECULE3	0.45	0.07	-0.03	A3	0.40	0.13
GEOG	MOLECULE4	0.45	0.05	-0.03	A3	0.06	0.12
DOGE	MOLECULE6	0.45	0.03	0.19	A2	0.18	0.31
OGSE	MOLECULE7	0.45	0.00	0.08	A1	0.25	0.18
PGEOGE	MOLECULE8	0.45	0.10	0.19	M	0.50	0.39
PG	MOLECULE9	0.05	0.00	-0.03	M	-0.56	-0.33
